# Adverse neonatal outcomes in small‐for‐gestational age twins identified using twin *vs* singleton growth charts: systematic review and meta‐analysis

**DOI:** 10.1002/uog.29298

**Published:** 2025-07-21

**Authors:** S. Sorrenti, D. Di Mascio, A. Khalil, F. D'Antonio, F. Zullo, E. D'Alberti, V. D'Ambrosio, I. Mappa, A. Giancotti, G. Rizzo

**Affiliations:** ^1^ Department of Maternal and Child Health and Urological Sciences Sapienza University of Rome Rome Italy; ^2^ Vascular Biology Research Centre, Molecular and Clinical Sciences Research Institute St George's University of London London UK; ^3^ Fetal Medicine Unit St George's University Hospitals NHS Foundation Trust, University of London London UK; ^4^ Center for Fetal Care and High‐Risk Pregnancy, Department of Obstetrics and Gynecology University of Chieti Chieti Italy; ^5^ Department of Obstetrics and Gynecology Fondazione Policlinico Tor Vergata University of Roma Tor Vergata Rome Italy

**Keywords:** fetal growth restriction, growth charts, multiple pregnancy, small‐for‐gestational age, twin charts, twin pregnancy, twins

## Abstract

**Objective:**

To evaluate the use of twin *vs* singleton growth charts for detecting small‐for‐gestational‐age (SGA) twins at risk of adverse neonatal outcomes.

**Methods:**

MEDLINE, EMBASE, CINAHL, Cochrane and Scopus databases were searched electronically from inception to May 2024. The primary outcome of this meta‐analysis was the risk of composite adverse neonatal outcome in SGA fetuses in a twin pregnancy diagnosed using twin or singleton charts. The secondary outcomes included: neonatal intensive care unit (NICU) admission, oxygen supplementation or continuous positive airway pressure, mechanical ventilation, respiratory distress syndrome, intraventricular hemorrhage, necrotizing enterocolitis, neonatal sepsis and neonatal mortality. Prospective and retrospective studies on neonatal outcomes of monochorionic or diamniotic twins diagnosed with SGA using both singleton and twin charts based on estimated fetal weight or birth weight were considered suitable for inclusion. Quality assessment of the included studies was performed using the Newcastle–Ottawa Scale for cohort studies. Random‐effects head‐to‐head meta‐analyses were used to analyze the data.

**Results:**

Six studies were included in the systematic review and five studies, including 10 554 twin pregnancies, were included in the meta‐analysis. The risk of composite adverse neonatal outcome (OR, 3.11 (95% CI, 1.83–5.26)) and that of most secondary outcomes was significantly higher in SGA fetuses diagnosed using twin charts compared with those diagnosed using singleton charts. Conversely, the risk of composite adverse neonatal outcome (OR, 1.22 (95% CI, 0.73–2.04)) and most secondary outcomes was similar when comparing SGA fetuses diagnosed using singleton charts *vs* non‐SGA fetuses diagnosed using twin charts, except for the risk of NICU admission, which was significantly higher in SGA fetuses diagnosed using singleton charts. When comparing non‐SGA fetuses diagnosed using twin charts *vs* non‐SGA fetuses diagnosed using singleton charts, the risk of composite adverse neonatal outcome was significantly lower when using twin charts (OR, 0.90 (95% CI, 0.83–0.97)). Finally, when comparing SGA *vs* non‐SGA fetuses diagnosed using singleton charts, there was no significant difference for the primary or secondary outcomes, except for a higher risk of NICU admission in the SGA group (OR, 1.54 (95% CI, 1.11–2.12)). Twin charts had lower sensitivity than singleton charts in predicting adverse neonatal outcome (14% (95% CI, 7–26%) *vs* 32% (95% CI, 24–41%)), but higher specificity (95% (95% CI, 86–98%) *vs* 71% (95% CI, 63–77%)).

**Conclusions:**

Twin charts increase the specificity but reduce the sensitivity for the detection of SGA compared with singleton charts. Nevertheless, twin charts detect cases at higher risk of adverse neonatal outcome, which may be the cases that require intervention. © 2025 The Author(s). *Ultrasound in Obstetrics & Gynecology* published by John Wiley & Sons Ltd on behalf of International Society of Ultrasound in Obstetrics and Gynecology.

## INTRODUCTION

Twin pregnancies are at higher risk of complications compared with singleton pregnancies, with growth disorders representing one of the most common causes of perinatal morbidity^1^, ^2^, ^3^.

The International Society of Ultrasound in Obstetrics and Gynecology (ISUOG) defines selective fetal growth restriction as a condition in which one fetus has an estimated fetal weight (EFW) below the 10^th^ percentile, with an intertwin weight discordance of 25% or more^4^.

To date, most international guidelines do not strongly recommend the use of twin‐specific growth charts to assess EFW in twin pregnancies. However, singleton charts classify nearly 30% of twin fetuses as small‐for‐gestational age (SGA), which is a disproportionately high percentage given that the all‐cause mortality rate of twin fetuses is around 1–2%^5^, ^6^. Though this might be partially explained by the increased surveillance in cases of SGA, the disproportion appears significant.

Slower growth velocity has been observed in twins, mainly in the third trimester. Whether the growth deceleration underlies physiological or pathological mechanisms remains controversial and the subject of long‐standing debates^5^. The major concern of maternal–fetal specialists regards the possibility that the reduced growth in the third trimester frequently observed in twin pregnancies may be caused by some degree of placental insufficiency, therefore requiring close surveillance^5^.

The correct choice of growth chart for the assessment of twin growth has been investigated by several studies that analyze the perinatal outcomes in SGA twins diagnosed using singleton charts compared with those in SGA twins diagnosed using twin charts^7^, and recent evidence suggests that twin charts are more accurate in predicting the risk of stillbirth in twin pregnancies complicated by SGA^6^, ^8^, ^9^. Conversely, the main concern when using twin charts might be related to the possible normalization of a pathological phenomenon.

The aim of this systematic review and meta‐analysis was to evaluate twin *vs* singleton growth charts in detecting SGA twins at risk of adverse neonatal outcomes.

## METHODS

### Protocol, information sources and literature search

This systematic review was performed according to a protocol designed *a priori* that is recommended for systematic reviews and meta‐analyses^10^, ^11^, ^12^. MEDLINE, EMBASE, CINAHL, Cochrane and Scopus databases were searched electronically from inception to May 2024, using combinations of the relevant medical subject heading (MeSH) terms, keywords and word variants for ‘twin charts’, ‘singleton charts’, ‘twin pregnancy’, ‘multiple pregnancy’, ‘perinatal outcome’, ‘neonatal outcome’, ‘small for gestational age’, ‘SGA’, ‘fetal growth restriction’ and ‘FGR’. Details regarding the search strategy are reported in Appendix S1. The search and selection criteria were restricted to studies published in the English language. Reference lists of relevant articles and reviews were searched manually for additional reports. Preferred Reporting Items for Systematic Review and Meta‐Analysis (PRISMA) guidelines were followed^13^, ^14^, ^15^. The study was registered with the PROSPERO database (registration number: CRD42024527397).

### Outcome measures, study selection and data collection

The primary outcome was risk of composite adverse neonatal outcome in SGA fetuses in a twin pregnancy diagnosed using twin or singleton charts (as defined in each included study). The secondary outcomes included: neonatal intensive care unit (NICU) admission, oxygen supplementation or continuous positive airway pressure (CPAP), mechanical ventilation, respiratory distress syndrome (RDS), intraventricular hemorrhage (IVH), necrotizing enterocolitis (NEC), neonatal sepsis and neonatal mortality. Outcomes were only included when they were reported in at least two studies.

All outcomes were also evaluated in cohorts of SGA fetuses diagnosed using singleton charts compared with non‐SGA fetuses diagnosed using twin charts. This comparison was performed to investigate the role of twin charts in identifying SGA fetuses at high risk of adverse neonatal outcome. Moreover, we also assessed these outcomes in non‐SGA twins classified using twin *vs* singleton charts. Finally, the outcome of SGA fetuses diagnosed using singleton charts was compared with that in non‐SGA fetuses classified using the same charts. Information was extracted regarding the twin and singleton charts that were used in the included studies. Each study cohort was evaluated using both singleton and twin charts, regardless of the use of each chart in clinical practice.

Prospective and retrospective studies investigating neonatal outcome in monochorionic or diamniotic twins diagnosed with SGA, using both singleton and twin charts based on EFW or birth weight, were considered suitable for inclusion. Only full‐text articles were considered eligible for inclusion. Case reports, case series with fewer than 10 cases, review articles, Letters to the Editor and editorials were excluded. Studies reporting outcomes of higher‐order pregnancies and studies published before 2000, as we considered that advances in the management of twin pregnancies make them less relevant, were also excluded.

Two authors (S.S., D.D.M.) reviewed all abstracts independently. Agreement regarding potential relevance was reached by consensus. Full‐text copies of articles deemed relevant were obtained, and the same two reviewers independently extracted relevant data regarding study characteristics and neonatal outcome. Inconsistencies were resolved through discussion among the reviewers until consensus was reached or through discussion with a third author (A.K.). Studies with data potentially overlapping with other studies already included in the analysis were excluded; the most recent study or that with more relevant data for the purpose of the study was considered for inclusion. Data not presented in the original publication were requested by e‐mail from the authors.

### Quality assessment and risk of bias

Quality assessment of the included studies was performed using the Newcastle–Ottawa Scale (NOS) for cohort studies. According to the NOS, each study is judged on three broad perspectives: selection of the study groups, comparability of the groups and ascertainment of the outcome of interest^16^. Assessment of the selection domain includes evaluation of the representativeness of the exposed cohort, selection of the non‐exposed cohort, ascertainment of exposure and demonstration that the outcome of interest was not present at the start of study. Assessment of the comparability domain includes evaluation of the comparability of cohorts based on design or analysis. Ascertainment of the outcome of interest includes evaluation of the type of assessment of the outcome of interest and length and adequacy of follow‐up. According to the NOS, a study can be awarded a maximum of one star for each numbered item within the selection and outcome domains, and a maximum of two stars can be given for comparability^16^.

### Statistical analysis

We used random‐effects head‐to‐head meta‐analysis to compare directly: (1) SGA fetuses diagnosed using twin *vs* singleton charts; (2) SGA fetuses diagnosed using singleton charts *vs* non‐SGA fetuses diagnosed using twin charts; (3) non‐SGA fetuses diagnosed using twin *vs* singleton charts; and (4) SGA *vs* non‐SGA fetuses both diagnosed using singleton charts, expressing the results as summary odds ratios (ORs) with 95% CIs for dichotomous outcomes and as mean differences with 95% CIs for continuous outcomes. In addition, diagnostic test accuracy analysis of the different charts in identifying cases of SGA complicated by composite adverse neonatal outcome, and other neonatal outcomes, was performed.

Statistical heterogeneity was evaluated using the *I* ^2^ statistic. *I* ^2^ values less than 25% were considered low, values between 25% and 50% were considered moderate and values greater than 50% were considered high. For each outcome, the total number of publications included in the meta‐analyses was less than 10. Therefore, we were unable to assess publication bias graphically, using funnel plots, or formally, using Egger's regression asymmetry test, because in such cases the power of the test would be too low to distinguish chance from real asymmetry^17^.

Statistical analyses were conducted using Review Manager (RevMan) software version 5.4.1 (Cochrane, London, UK) and STATA version 18 (StataCorp LLC., College Station, TX, USA).

## RESULTS

### Study selection and characteristics

**Figure 1:**
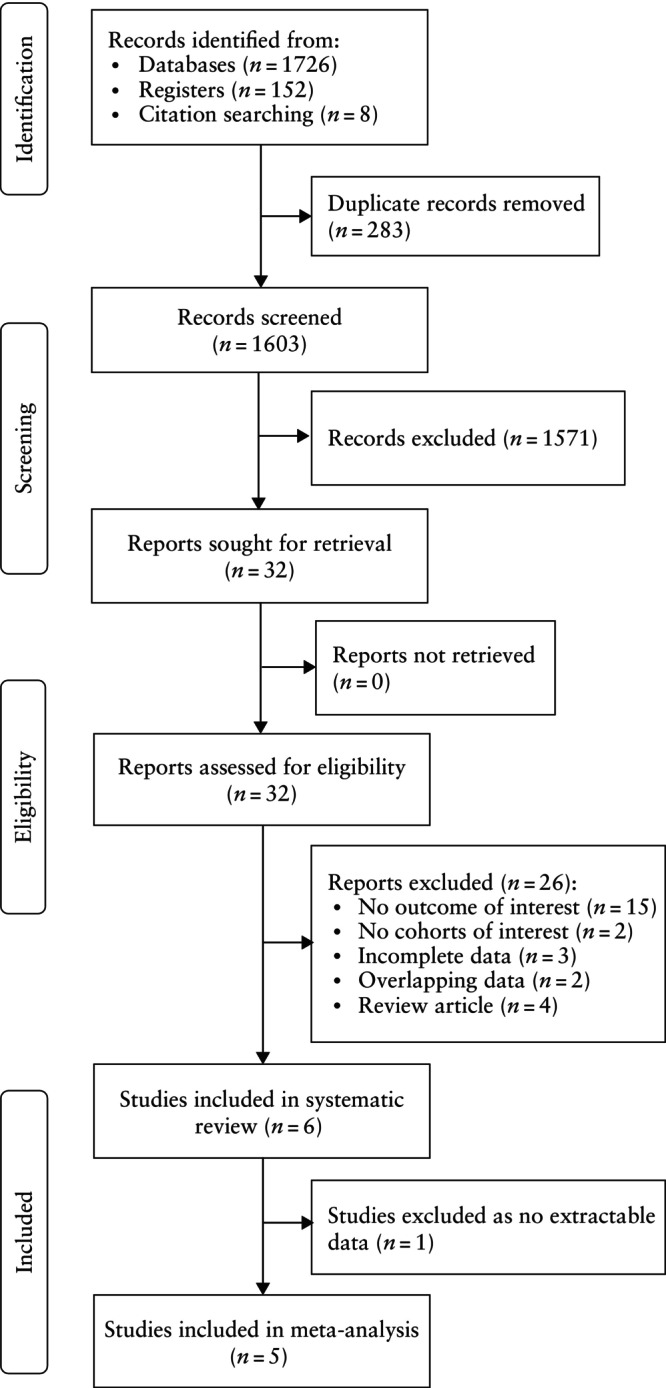
Flowchart summarizing inclusion of studies in systematic review and meta‐analysis.

The literature search and other methods identified 1886 articles, of which 32 were assessed with respect to their eligibility for inclusion and six studies were included in the systematic review (Figure 1, Tables 1 and S1)^18^, ^19^, ^20^, ^21^, ^22^, ^23^. However, only five studies were included in the meta‐analysis^18^, ^19^, ^20^, ^21^, ^23^ as one study did not report extractable data^22^.

**Table 1:** General characteristics of studies included in systematic review

Study	Study period	Country	Chorionicity	Sample size	Gestational age at delivery (weeks)	Study group
Briffa (2022)^18^	2007–2020	UK	DC and MC	913 pregnancies (723 DC, 190 MC); 1826 fetuses	37.0 (36.1–37.4)†	Only live births
Giorgione (2021)^19^	2000–2020	UK	DC and MC	1740 pregnancies (1349 DC, 391 MC); 3480 fetuses	36.7 (34.6–37.4)†	All twins (including perinatal deaths)
Nowacka (2021)^20^	2005–2015	Poland	DC and MC	322 pregnancies (247 DC, 75 MC); 644 fetuses	92% delivered > 32	Only live births
Lin (2021)^21^	2012–2020	China	DC and MC	3027 pregnancies (2485 DC, 542 MC); 6054 fetuses	35.8 ± 1.8†	Only live births
Shea (2021)^22^	2004–2019	USA	DC	730 pregnancies; 1460 fetuses	≥ 32	Only live births
Mendez‐Figueroa (2018)^23^	1989–2004	USA	DC and MC	4552 pregnancies; 7673 fetuses*	79.3% delivered > 32	Only live births

Only first author is given for each study.

All studies were retrospective.

* Only available data are reported.

† Data are given as median (interquartile range) or mean ± SD.

DC, dichorionic; MC, monochorionic.

These five studies included 10 554 twin pregnancies, of which 4804 were dichorionic, 1198 were monochorionic and chorionicity was unspecified in 4552. All included studies were retrospective. Data regarding twin or singleton charts used^24^, ^25^, ^26^, ^27^, ^28^, ^29^, ^30^, ^31^, ^32^, ^33^, rate of SGA detected using the different charts and the definition of composite adverse neonatal outcome used for each study are reported in Table 2.

**Table 2:** Growth charts used and definition of composite adverse neonatal outcome in included studies

Study	SGA by singleton chart (%)	SGA by twin chart (%)	Singleton chart used	Twin chart used	Composite adverse neonatal outcome
Briffa (2022)^18^	33.3	5.9	Nicolaides *et al*.^24^	Stirrup *et al*.^25^	Oxygen supplementation or CPAP for < 72 h, hypoglycemia, hypocalcemia, hyperbilirubinemia, IVH, NEC, BPD, RDS, need for mechanical ventilation, neonatal death
Giorgione (2021)^19^	34.8	8.3	Nicolaides *et al*.^24^	Stirrup *et al*.^25^	NA
Nowacka (2021)^20^	20.0	10.0	Working Group on Fetal Biometric Charts^26^	Working Group on Fetal Biometric Charts^26^	5‐min Apgar score < 8, need for intubation, need for CPAP or mechanical ventilation, NICU admission, IVH Grade III or IV, NEC, neonatal pneumonia or inborn infection, neonatal death within 28 days after birth
Lin (2021)^21^	33.1	7.3	Population‐based birth‐weight percentiles for Chinese singletons^28^	Population‐based birth‐weight percentiles for Chinese twins^29^	Hypoxic ischemic encephalopathy, NEC, intracranial hemorrhage, BPD, sepsis, neonatal death
Shea (2021)^22^	16.9	8.8	Hadlock singleton growth reference^30^	NICHD twin‐specific growth reference^31^	Mild neonatal morbidity: oxygen supplementation or CPAP for < 72 h, hypoglycemia, hypocalcemia, hyperbilirubinemia, IVH Grade I or II. Severe neonatal morbidity: NEC Grade 2A, IVH Grade III or IV, BPD, need for mechanical ventilation, neonatal death
Mendez‐Figueroa (2018)^23^	33	4	United States fetal growth charts by Alexander *et al*.^32^	Ananth *et al*.^33^	5‐min Apgar score < 4, RDS, need for mechanical ventilation, IVH Grade III or IV, NEC Grade 2 or 3, neonatal sepsis, periventricular leukomalacia, confirmed seizure, stillbirth, neonatal death

Only first author is given for each study.

BPD, bronchopulmonary dysplasia; CPAP, continuous positive airway pressure; IVH, interventricular hemorrhage; NA, not available; NEC, necrotizing enterocolitis; NICHD, National Institute of Child Health and Human Development; NICU, neonatal intensive care unit; RDS, respiratory distress syndrome; SGA, small‐for‐gestational age.

All studies except one^19^ reported neonatal data only among live births. Three studies used EFW‐based twin charts to assess growth in the included cases^18^, ^19^, ^20^, whereas two studies used birth‐weight‐based twin charts^21^, ^23^. For those that used ultrasound‐based charts, the assessment considered in the analysis was the last assessment before delivery, in the third trimester.

The results of the quality assessment of the included studies using the NOS are presented in Table 3. Overall, the quality of the included studies was good; 3/5 studies were given the maximum score for selection, 3/5 were given the maximum score for comparability and 4/5 were given the maximum score for outcome representation.

**Table 3:** Quality assessment of included studies according to Newcastle–Ottawa Scale for cohort studies

Author	Selection	Comparability	Outcome
Briffa (2022)^18^	★★★	★	★★★
Giorgione (2021)^19^	★★	★★	★★
Nowacka (2021)^20^	★★★	★★	★★★
Lin (2021)^21^	★★★	★★	★★★
Mendez‐Figueroa (2018)^23^	★★	★	★★★

Only first author is given for each study.

### Synthesis of results

#### *SGA diagnosed using twin charts* vs *SGA diagnosed using singleton charts*

**Figure 2:**
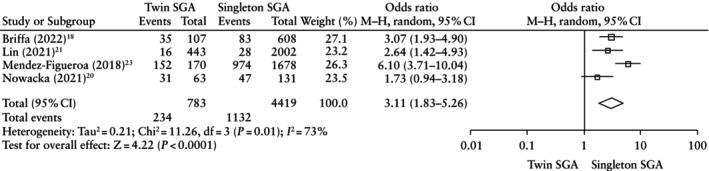
Forest plot showing risk of composite adverse neonatal outcome in small‐for‐gestational‐age (SGA) fetuses diagnosed using twin *vs* singleton charts. Only first author is given for each study. M–H, Mantel–Haenszel.

The risk of composite adverse neonatal outcome was significantly higher in SGA fetuses diagnosed using twin charts compared with those diagnosed using singleton charts (OR, 3.11 (95% CI, 1.83–5.26); *P* < 0.0001) (Figure 2, Table 4). Similarly, most other adverse neonatal outcomes were significantly higher in cases classified as SGA using twin charts, such as NICU admission (OR, 3.77 (95% CI, 2.46–5.77); *P* < 0.001); need for mechanical ventilation (OR, 2.39 (95% CI, 1.83–3.12); *P* < 0.001); RDS (OR, 2.25 (95% CI, 1.83–2.75); *P* < 0.001); IVH (OR, 3.31 (95% CI, 1.74–6.29); *P* < 0.001); NEC

**Table 4:** Neonatal outcomes in small‐for‐gestational‐age fetuses diagnosed using twin *vs* singleton charts

Outcome	Studies	Raw proportions (*n*/*N* (%) *vs n*/*N* (%))	Pooled OR (95% CI)	*I* ^2^ (%)	*P*
CANO	4^18^, ^20^, ^21^, ^23^	234/783 (29.9) *vs* 1132/4419 (25.6)	3.11 (1.83–5.26)	73	< 0.0001
NICU admission	3^19^, ^20^, ^21^	570/774 (73.6) *vs* 1324/3305 (40.1)	3.77 (2.46–5.77)	78	< 0.001
Oxygen supplementation or CPAP	2^18^, ^20^	19/170 (11.2) *vs* 39/739 (5.3)	1.44 (0.77–2.70)	0	0.25
Need for mechanical ventilation	4^18^, ^20^, ^21^, ^23^	144/919 (15.7) *vs* 411/5256 (7.8)	2.39 (1.83–3.12)	26	< 0.001
RDS	3^18^, ^21^, ^23^	152/856 (17.8) *vs* 516/5126 (10.1)	2.25 (1.83–2.75)	0	< 0.001
IVH	4^18^, ^20^, ^21^, ^23^	15/887 (1.7) *vs* 26/4773 (0.5)	3.31 (1.74–6.29)	0	< 0.001
NEC	3^18^, ^20^, ^23^	4/217 (1.8) *vs* 8/1630 (0.5)	4.16 (1.18–14.63)	0	0.03
Neonatal sepsis	3^20^, ^21^, ^23^	108/660 (16.4) *vs* 869/4263 (20.4)	2.47 (1.82–3.37)	0	< 0.001
Neonatal mortality	4^18^, ^20^, ^21^, ^23^	32/878 (3.6) *vs* 53/4747 (1.1)	3.81 (2.43–5.99)	0	< 0.001

CANO, composite adverse neonatal outcome; CPAP, continuous positive airway pressure; IVH, intraventricular hemorrhage; NEC, necrotizing enterocolitis; NICU, neonatal intensive care unit; OR, odds ratio; RDS, respiratory distress syndrome.

(OR, 4.16 (95% CI, 1.18–14.63); *P* = 0.03); neonatal sepsis (OR, 2.47 (95% CI, 1.82–3.37); *P* < 0.001); and neonatal mortality (OR, 3.81 (95% CI, 2.43–5.99); *P* < 0.001). Oxygen supplementation or CPAP did not differ significantly among the two groups (OR, 1.44 (95% CI, 0.77–2.70); *P* = 0.25).

In agreement with these results from the meta‐analysis, the study of Shea *et al*.^22^ showed that the SGA cases diagnosed using twin charts had a higher risk of mild composite adverse neonatal outcome compared with singleton charts (adjusted OR (aOR), 2.03 (95% CI, 1.00–4.14); *P* = 0.034). Severe composite neonatal outcome was found to be increased in the former group, but significance was not reached (aOR, 3.70 (95% CI, 0.72–18.90); *P* = 0.11).

#### *SGA diagnosed using singleton charts* vs *non‐SGA diagnosed using twin charts*

**Figure 3:**
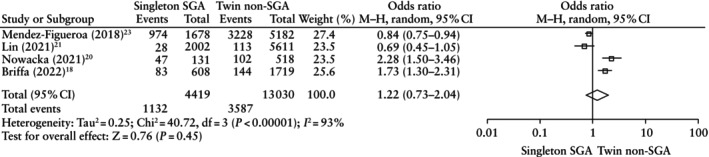
Forest plot showing risk of composite adverse neonatal outcome in small‐for‐gestational‐age (SGA) fetuses diagnosed using singleton charts *vs* non‐SGA fetuses diagnosed using twin charts. Only first author is given for each study. M–H, Mantel–Haenszel.

The risk of composite adverse neonatal outcome was similar when comparing SGA fetuses diagnosed using singleton charts *vs* non‐SGA fetuses diagnosed using twin charts (OR, 1.22 (95% CI, 0.73–2.04); *P* = 0.45) (Figure 3). When considering the secondary outcomes, there was no significant difference between the two groups in terms of oxygen supplementation or CPAP (OR, 1.18 (95% CI, 0.75–1.85); *P* = 0.47); need for mechanical ventilation (OR, 1.12 (95% CI, 0.63–1.99); *P* = 0.70); RDS (OR, 0.82 (95% CI, 0.53–1.27); *P* = 0.38); IVH (OR, 1.12 (95% CI, 0.39–3.19); *P* = 0.83); NEC (OR, 2.01 (95% CI, 0.04–103.68); *P* = 0.73); neonatal sepsis (OR, 0.93 (95% CI, 0.58–1.50); *P* = 0.77); or neonatal mortality (OR, 1.58 (95% CI, 0.72–3.50); *P* = 0.26). Only the risk of NICU admission was significantly higher in the SGA diagnosed using singleton charts group (OR, 1.55 (95% CI, 1.20–2.01); *P* < 0.001) (Table 5).

**Table 5:** Neonatal outcomes in small‐for‐gestational‐age (SGA) fetuses diagnosed using singleton charts *vs* non‐SGA fetuses diagnosed using twin charts

Outcome	Studies	Raw proportions (*n*/*N* (%) *vs n*/*N* (%))	Pooled OR (95% CI)	*I* ^2^ (%)	*P*
CANO	4^18^, ^20^, ^21^, ^23^	1132/4419 (25.6) *vs* 3587/13 030 (27.5)	1.22 (0.73–2.04)	93	0.45
NICU admission	3^19^, ^20^, ^21^	1324/3305 (40.1) *vs* 2991/9195 (32.5)	1.55 (1.20–2.01)	84	< 0.001
Oxygen supplementation or CPAP	2^18^, ^20^	39/739 (5.3) *vs* 115/2237 (5.1)	1.18 (0.75–1.85)	27	0.47
Need for mechanical ventilation	4^18^, ^20^, ^21^, ^23^	411/5256 (7.8) *vs* 1805/15 058 (12.0)	1.12 (0.63–1.99)	93	0.70
RDS	3^18^, ^21^, ^23^	516/5126 (10.1) *vs* 2193/14 540 (15.1)	0.82 (0.53–1.27)	91	0.38
IVH	4^18^, ^20^, ^21^, ^23^	26/4773 (0.5) *vs* 105/13 743 (0.8)	1.12 (0.39–3.19)	74	0.83
NEC	3^18^, ^20^, ^23^	8/1630 (0.5) *vs* 51/4908 (1.0)	2.01 (0.04–103.68)	84	0.73
Neonatal sepsis	3^20^, ^21^, ^23^	869/4263 (20.4) *vs* 2837/12 314 (23.0)	0.93 (0.58–1.50)	58	0.77
Neonatal mortality	4^18^, ^20^, ^21^, ^23^	53/4747 (1.1) *vs* 148/13 726 (1.1)	1.58 (0.72–3.50)	61	0.26

CANO, composite adverse neonatal outcome; CPAP, continuous positive airway pressure; IVH, intraventricular hemorrhage; NEC, necrotizing enterocolitis; NICU, neonatal intensive care unit; OR, odds ratio; RDS, respiratory distress syndrome.

#### *Non‐SGA diagnosed using twin charts* vs *non‐SGA diagnosed using singleton charts*

**Figure 4:**
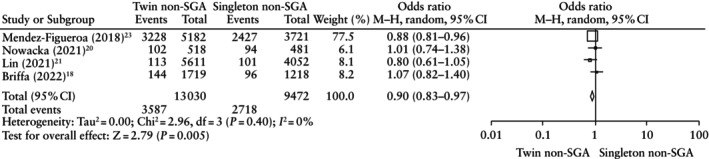
Forest plot showing risk of composite adverse neonatal outcome in non‐small‐for‐gestational‐age (SGA) fetuses diagnosed using twin charts *vs* singleton charts. Only first author is given for each study. M–H, Mantel–Haenszel.

The risk of composite adverse neonatal outcome was significantly lower for non‐SGA fetuses diagnosed using twin charts compared with non‐SGA fetuses diagnosed using singleton charts (OR, 0.90 (95% CI, 0.83–0.97); *P* = 0.005) (Figure 4). Similarly, the risk of RDS and the need for mechanical ventilation were also significantly lower in non‐SGA fetuses diagnosed using twin charts (OR, 0.82 (95% CI, 0.75–0.89); *P* < 0.001 and OR, 0.80 (95% CI, 0.75–0.87); *P* < 0.001, respectively). The risk of neonatal sepsis was also lower in the group diagnosed using twin charts (OR, 0.89 (95% CI, 0.82–0.96); *P* = 0.002). The remaining neonatal outcomes were similar between the two groups (Table 6).

**Table 6:** Neonatal outcomes in non‐small‐for‐gestational‐age fetuses diagnosed using twin *vs* singleton charts

Outcome	Studies	Raw proportions (*n*/*N* (%) *vs n*/*N* (%))	OR (95% CI)	*I* ^2^ (%)	*P*
CANO	4^18^, ^20^, ^21^, ^23^	3587/13 030 (27.5) *vs* 2718/9472 (28.7)	0.90 (0.83–0.97)	0	0.005
NICU admission	3^19^, ^20^, ^21^	2991/9195 (32.5) *vs* 2243/6708 (33.4)	0.95 (0.89–1.02)	0	0.15
Oxygen supplementation or CPAP	2^18^, ^20^	115/2237 (5.1) *vs* 99/1699 (5.8)	0.97 (0.73–1.28)	0	0.81
Need for mechanical ventilation	4^18^, ^20^, ^21^, ^23^	1805/15 058 (12.0) *vs* 1552/10 882 (14.3)	0.80 (0.75–0.87)	0	< 0.001
RDS	3^18^, ^21^, ^23^	2193/14 540 (15.1) *vs* 1841/10 400 (17.7)	0.82 (0.75–0.89)	11	< 0.001
IVH	4^18^, ^20^, ^21^, ^23^	105/13 743 (0.8) *vs* 95/10 014 (0.9)	0.80 (0.61–1.06)	0	0.12
NEC	3^18^, ^20^, ^23^	51/4908 (1.0) *vs* 47/3554 (1.3)	0.75 (0.50–1.12)	N/A	0.16
Neonatal sepsis	3^20^, ^21^, ^23^	2837/12 314 (23.0) *vs* 2083/8777 (23.7)	0.89 (0.82–0.96)	0	0.002
Neonatal mortality	4^18^, ^20^, ^21^, ^23^	148/13 726 (1.1) *vs* 130/10 017 (1.3)	0.83 (0.65–1.05)	0	0.12

CANO, composite adverse neonatal outcome; CPAP, continuous positive airway pressure; IVH, intraventricular hemorrhage; N/A, not applicable; NEC, necrotizing enterocolitis; NICU, neonatal intensive care unit; OR, odds ratio; RDS, respiratory distress syndrome.

#### *SGA* vs *non‐SGA diagnosed using singleton charts*

**Figure 5:**
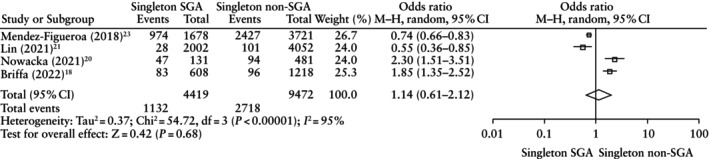
Forest plot showing risk of composite adverse neonatal outcome in small‐for‐gestational age (SGA) *vs* non‐SGA fetuses diagnosed using singleton charts. Only first author is given for each study. M–H, Mantel–Haenszel.

When comparing SGA *vs* non‐SGA fetuses diagnosed using singleton charts, there was no significant difference for the primary and secondary outcomes, except for a higher risk of NICU admission in the SGA group (OR, 1.54 (95% CI, 1.11–2.12); *P* = 0.009) (Figure 5, Table 7).

**Table 7:** Neonatal outcomes in small‐for‐gestational‐age (SGA) *vs* non‐SGA fetuses diagnosed using singleton charts

Outcome	Studies	Raw proportions (*n*/*N* (%) *vs n*/*N* (%))	OR (95% CI)	*I* ^2^ (%)	*P*
CANO	4^18^, ^20^, ^21^, ^23^	1132/4419 (25.6) *vs* 2718/9472 (28.7)	1.14 (0.61–2.12)	95	0.68
NICU admission	3^19^, ^20^, ^21^	1324/3305 (40.1) *vs* 2243/6708 (33.4)	1.54 (1.11–2.12)	89	0.009
Oxygen supplementation or CPAP	2^18^, ^20^	39/739 (5.3) *vs* 99/1699 (5.8)	1.15 (0.73–1.80)	24	0.56
Need for mechanical ventilation	4^18^, ^20^, ^21^, ^23^	411/5256 (7.8) *vs* 1552/10 882 (14.3)	1.02 (0.53–1.98)	95	0.95
RDS	3^18^, ^21^, ^23^	516/5126 (10.1) *vs* 1841/10 400 (17.7)	0.74 (0.43–1.26)	94	0.26
IVH	4^18^, ^20^, ^21^, ^23^	26/4773 (0.5) *vs* 95/10 014 (0.9)	1.03 (0.31–3.44)	78	0.96
NEC	3^18^, ^20^, ^23^	8/1630 (0.5) *vs* 47/3554 (1.3)	1.70 (0.03–109.20)	86	0.80
Neonatal sepsis	3^20^, ^21^, ^23^	869/4263 (20.4) *vs* 2083/8777 (23.7)	0.83 (0.50–1.37)	60	0.47
Neonatal mortality	4^18^, ^20^, ^21^, ^23^	53/4747 (1.1) *vs* 130/10 017 (1.3)	1.29 (0.60–2.79)	59	0.52

CANO, composite adverse neonatal outcome; CPAP, continuous positive airway pressure; IVH, intraventricular hemorrhage; NEC, necrotizing enterocolitis; NICU, neonatal intensive care unit; OR, odds ratio; RDS, respiratory distress syndrome.

### Diagnostic test accuracy of twin and singleton charts

The evaluation of diagnostic performance in identifying cases of SGA complicated by composite adverse neonatal outcome, neonatal death or NICU admission is presented in Table 8.

**Table 8:** Diagnostic test accuracy of twin and singleton charts

	Twin charts		Singleton charts	
Outcome	Sensitivity (95% CI)	Specificity (95% CI)	Sensitivity (95% CI)	Specificity (95% CI)
CANO	0.14 (0.07–0.26)	0.95 (0.86–0.98)	0.32 (0.24–0.41)	0.71 (0.63–0.77)
Neonatal death	0.25 (0.14–0.40)	0.94 (0.91–0.95)	0.32 (0.21–0.44)	0.70 (0.65–0.75)
NICU admission	0.19 (0.14–0.25)	0.96 (0.94–0.98)	0.37 (0.31–0.43)	0.73 (0.65–0.80)

CANO, composite adverse neonatal outcome; NICU, neonatal intensive care unit.

## DISCUSSION

### Summary of the main findings

The findings from this study show that the risks of composite adverse neonatal outcome and most of the secondary outcomes were significantly higher in SGA fetuses diagnosed using twin charts compared to those diagnosed using singleton charts, whereas these outcomes were similar when comparing SGA fetuses diagnosed using singleton charts with non‐SGA fetuses diagnosed using twin charts (except for NICU admission). Moreover, the risk of composite adverse neonatal outcome was significantly lower when comparing non‐SGA fetuses diagnosed using twin *vs* singleton charts. Additionally, the risk of neonatal complications was similar when comparing SGA *vs* non‐SGA fetuses diagnosed using singleton charts. The accuracy analysis showed that twin charts had lower sensitivity and higher specificity compared with singleton charts in predicting composite adverse neonatal outcome, neonatal death and NICU admission.

### Clinical and research implications

Regardless of chorionicity, twin pregnancy is associated with a higher rate of perinatal morbidity and mortality compared with singleton pregnancy, so cases require closer sonographic surveillance to detect any change in growth and hemodynamics in one or both twins^4^. It is well‐known that twins have a slower fetal growth trend compared with singletons, with this difference becoming most relevant in the second half of pregnancy, and leading to twins usually being smaller at birth than singletons^5^. Therefore, the main challenge when evaluating fetal growth in twins is to distinguish whether the divergent growth trajectories, compared with those seen in singletons, are caused by a pathological placental insufficiency leading to impaired growth or by a physiological adaptation of the placenta to supply the nutritional and metabolic demands of two rapidly evolving organisms, rather than one, as an evolutionary mechanism to increase the chances of perinatal survival and reduce excessive distention of the uterine walls^5^. At present, there is no strong evidence to support either of the two proposed etiologies^5^.

This longstanding debate regarding the pathophysiology of fetal growth deceleration in the second half of twin pregnancy has also led to another important clinical challenge, namely the choice of using singleton *vs* twin‐specific growth charts to monitor fetal growth in twins, with those supporting the theory of decreased growth velocity as an underlying pathological condition usually supporting the use of singleton charts. The use of singleton charts would certainly provide a standardized benchmark, allowing the evaluation of fetal size irrespective of the type of pregnancy (i.e. singleton or twin) and avoiding arbitrary adjustments for abnormal growth curves in twins^34^, ^35^. When using singleton charts, over 30% of twins can be diagnosed as SGA, which is significantly higher than that observed when using twin charts (Table 2). On the other hand, considering fetal growth deceleration as a physiological adaptation generally leads to reliance on twin‐specific growth charts, with the purpose of decreasing the ‘overdiagnosis’ of SGA in twins and the subsequent clinical and psychological consequences associated with such a diagnosis. It is argued that this approach is justified also when considering the evidence that twin fetuses classified as SGA present signs of placental insufficiency less frequently on histological examination, compared with singletons classified as SGA^36^, ^37^.

Traditionally, twin pregnancies are monitored according to growth standards established for singleton pregnancies. However, studies have reported that use of twin‐specific charts might reduce the rate of false‐positive cases with pathological growth restriction^38^, ^39^. In this scenario, most international societies do not provide any specific recommendation on which growth chart is preferred^40^, ^41^, ^42^, ^43^, although the most recent guidelines seem to endorse the use of twin growth charts to avoid overdiagnosis of impaired growth disorders^44^.

The findings of this study suggest that the use of twin charts should be evaluated in larger studies for their applicability in clinical practice. Our findings are concordant with recent literature^5^, ^6^, ^7^, which supports the use of twin charts for the assessment of fetal growth in twin pregnancies, due to a putatively higher specificity of twin charts in identifying high‐risk cases that require close surveillance and possibly active intervention. In fact, our data showed that adverse perinatal outcomes were significantly more frequent in SGA fetuses diagnosed using twin charts compared with those diagnosed using singleton charts, and the risks of adverse perinatal outcomes were similar when comparing SGA fetuses diagnosed using singleton charts *vs* non‐SGA fetuses diagnosed using twin charts, with the exception of NICU admission, which, in our opinion, may also reflect the common clinical practice of admitting twin neonates classed as SGA to the NICU for close perinatal monitoring more than admittance for an objective clinical need.

We acknowledge that, when adjusting for the true growth trajectory of twin fetuses, twin charts appear more restrictive in the diagnosis of SGA, therefore including only the true small fetuses that are naturally at an increased risk of adverse outcome, as demonstrated in our analysis. This process has the potential for reducing the false‐positive diagnoses of SGA and increasing the specificity. However, it reduces the sensitivity for adverse outcomes, using a broader and more inclusive definition of SGA. In fact, the accuracy analysis showed a low sensitivity of twin charts in the prediction of composite adverse neonatal outcome, neonatal death and NICU admission, but high specificity. The sensitivity was found to be higher in singleton charts (32% for composite adverse neonatal outcome, 32% for neonatal death and 37% for NICU admission), but with a lower specificity (less than 75% for all evaluated outcomes) compared with twin charts. This might be indicative of the studies being underpowered to evaluate adverse outcomes in a cohort of SGA twins, which is a limitation in the interpretation of these results.

On the other hand, the comparison of SGA fetuses diagnosed using singleton charts with non‐SGA fetuses diagnosed using twin charts aimed to demonstrate the bias that exists in the definition of SGA using the different charts. The similar outcomes observed in this comparison might be related to the failure to diagnose cases at risk of adverse outcomes using twin charts or to the over‐comprehensive diagnosis of SGA using singleton charts. This concept was addressed in the comparison of non‐SGA diagnosis using the two different charts, which demonstrated that those classified as non‐SGA using twin charts did not experience higher rates of adverse neonatal outcomes. The lower accuracy of singleton charts in detecting high‐risk SGA in twin pregnancies was also demonstrated by the similar risks of adverse neonatal outcomes when comparing SGA *vs* non‐SGA fetuses diagnosed using singleton charts.

However, as mentioned above, these data reflect the clinical practice in centers of high‐expertise, which therefore suggests limited applicability on a larger scale. We speculate that a large‐cluster randomized controlled trial regarding the use of twin or singleton charts for growth assessment may investigate properly whether twin charts can be safely used in clinical practice to reduce the morbidity associated with iatrogenic prematurity, without increasing the risk of stillbirth due to reduced surveillance or delayed delivery. In addition, further studies regarding the mechanisms underlying the decelerated growth observed in twins in the late second trimester and robust clinical data regarding long‐term outcomes are needed in order to consider the adoption of twin‐specific growth charts in clinical practice.

### Strengths and limitations

To the best of our knowledge, this is the first meta‐analysis comparing the risk of composite adverse neonatal outcome and overall perinatal morbidity occurring in SGA twins diagnosed using twin *vs* singleton growth charts. The robust methodology, thorough literature search and the large number of both the cases included and the outcomes assessed represent additional strengths of this study.

The retrospective nature of the included studies, use of different twin and singleton growth charts for the assessment of fetal growth and the lack of standardized care for cases diagnosed with SGA in different clinical settings represent the main limitations of this study. In particular, regarding the last of these, iatrogenic preterm birth represents a significant, potential bias that may influence neonatal outcomes. Moreover, based on the available data from the included studies, we could not address one of the most important issues in the management of SGA twin pregnancies, namely the risk of stillbirth when using twin or singleton charts, as all included studies were underpowered with regard to this outcome. In fact, the majority of the studies reported perinatal outcomes only among live births and one study included stillbirth in the definition of composite adverse neonatal outcome^23^. We acknowledge that this is a major limitation and may have influenced the primary outcome. However, the rate of stillbirth reported in this study was low (less than 2% among SGA cases diagnosed using singleton charts and less than 6% in those diagnosed using twin charts)^23^. We also acknowledge that the rate of stillbirth in twin pregnancies complicated by SGA might be biased by the increased surveillance and targeted timing of delivery in different clinical practices, in which most of the studies were conducted in high‐expertise centers. Therefore, the included studies may not represent the overall situation in smaller, non‐academic centers.

Lastly, as a major limitation, we were not able to adjust the outcomes for gestational age at delivery, which we acknowledge as one of the most important determinants of adverse perinatal outcome.

### Conclusions

The current literature is still lacking on the mechanisms underlying the different growth pathways in twin pregnancies compared with those in singletons. The use of twin growth charts reduces the sensitivity in the identification of SGA fetuses; however, these charts increase the specificity and positive‐predictive value. Further randomized controlled trials are needed to investigate whether twin charts might reduce the rate of unnecessary iatrogenic prematurity and parental anxiety by limiting close antenatal surveillance and active management to only those cases at higher risk of complications.

## Supporting information


**Appendix S1** Search strategy.
**Table S1** Excluded studies and reason for exclusion.

## Data Availability

The data that support the findings of this study are available from the corresponding author upon reasonable request.
