# Supplementary material for: Adverse neonatal outcomes in small‐for‐gestational age twins identified using twin vs singleton growth charts: systematic review and meta‐analysis
Source: Ultrasound Obstet Gynecol. 2025 Jul 21;66(6):706–15. doi: 10.1002/uog.29298 (PMC12671938; doi:10.1002/uog.29298)
Supplement: Supplementary file 1 — Appendix S1 Search strategy. Table S1 Excluded studies and reason for exclusion. [file UOG-66-706-s001.docx]

**Table S1** Excluded studies and reason for exclusion

| Authors | Year | Title | Reason for exclusion |
| --- | --- | --- | --- |
| Martinez-Varea | 2024 | Association of fetal growth restriction and stillbirth in twin compared with singleton pregnancies | Not outcome of interest |
| Dekalo | 2023 | Fetal growth restriction, neonatal morbidity and placental pathology in dichorionic twins-a comparison of twin-specific versus singleton growth charts | Incomplete data |
| Dall’Asta | 2023 | Clinical implementation of twin-specific growth charts: still more work to do | Review article |
| Hiersch | 2022 | Should twin-specific growth charts be used to assess fetal growth in twin pregnancies? | Review article |
| Kalafat | 2022 | Assessment of fetal growth in twins: Which method to use? | Review article |
| Melamed | 2022 | Fetal Growth in Twin Pregnancies and the Choice of Growth Chart | Review article |
| Hugh | 2021 | Fetal growth in twins: twin-specific versus singleton customised growth charts and stillbirth risk | Only abstract-not full text |
| Hiersch | 2021 | Patterns of discordant growth and adverse neonatal outcomes in twins | Not outcome of interest |
| Lyu | 2021 | The association between maternal complications and small for gestational age in twin pregnancies using singleton and twin birth weight references | Not outcome of interest (only maternal data) |
| Briffa | 2021 | Twin chorionicity-specific population birth-weight charts adjusted for estimated fetal weight | Not outcome of interest |
| Kalafat | 2020 | The Association Between Hypertension in Pregnancy and Preterm Birth with Fetal Growth Restriction in Singleton and Twin Pregnancy: Use of Twin Versus Singleton Charts | Overlapping data with included studies |
| Giorgione | 2020 | Are Twin Pregnancies Complicated by Weight Discordance or Fetal Growth Restriction at Higher Risk of Preeclampsia? | Not outcome of interest |
| Horst | 2020 | Outcome dependent twin growth curves based on birth weight percentiles for Polish population | Not outcome of interest |
| Francis | 2019 | Fetal growth in twins: comparison of twin-specific STORK and singleton customised GROW charts | Abstract- not full text |
| Proctor | 2019 | Association between hypertensive disorders and fetal growth restriction in twin compared with singleton gestations | No cohort of interest |
| Kalafat | 2019 | Predictive accuracy of Southwest Thames Obstetric Research Collaborative (STORK) chorionicity-specific twin growth charts for stillbirth: a validation study | Overlapping data with included studies |
| Hiersch | 2018 | Differences in fetal growth patterns between twins and singletons | Not outcome of interest |
| Cordiez | 2017 | Impact of customized growth curves on screening for small for gestational age twins | Not outcome of interest |
| Stirrup | 2015 | Fetal growth reference ranges in twin pregnancy: analysis of the Southwest Thames Obstetric Research Collaborative (STORK) multiple pregnancy cohort | Not outcome of interest |
| Atoof | 2015 | Two-Year Comparison of Growth Indices of Twins with Dissimilar Weight at Birth (Low Birth Weight vs. Normal Twin) | Not outcome of interest |
| Odibo | 2013 | Customized growth charts for twin gestations to optimize identification of small-for-gestational age fetuses at risk of intrauterine fetal death | No cohort of interest |
| Dias | 2010 | First-trimester ultrasound dating of twin pregnancy: are singleton charts reliable? | No outcome of interest |
| Joseph | 2009 | An Outcome-based Approach for the Creation of Fetal Growth Standards: Do Singletons and Twins Need Separate Standards? | No outcome of interest |
| Gielen | 2008 | Twin-Specific Intrauterine ‘Growth’ Charts Based on Cross-Sectional Birthweight Data | No outcome of interest |
| Min | 2000 | Birth weight references for twins | No outcome of interest |
| Grumbach | 1986 | Twin and singleton growth patterns compared using US | No outcome of interest |

**Appendix S1** Search strategy

**Pubmed:**

|  |  |  |
| --- | --- | --- |
| #1 | "pregnancy, twin"[MeSH Terms] OR "pregnancy, multiple"[MeSH Terms] OR "Twins"[MeSH Terms] OR "twinning, embryonic"[MeSH Terms] | 37153 |
| #4 | (("twins"[MeSH Terms] OR "twins"[All Fields] OR "twin"[All Fields]) AND "pregancy"[Title/Abstract]) OR "multiple pregnancy"[Title/Abstract] OR "twins"[Title/Abstract] OR "twin study"[Title/Abstract] OR "multiple birth"[Title/Abstract] | 40427 |
| #15 | "Fetal Weight"[MeSH Terms] OR "Birth Weight"[MeSH Terms] OR "Gestational Age"[MeSH Terms] | 124421 |
| #18 | (#1 OR #4) AND #15 | 123271 |
| #5 | (((growth chart) OR (birthweight chart)) OR (fetal weight chart)) OR (weight chart) | 16543 |
| #19 | #18 AND #5 | 1776 |
| #13 | chart[Title/Abstract] | 83709 |
| **#21** | **#19 AND #13** | **770** |

Full strategy:

((("pregnancy, twin"[MeSH Terms] OR "pregnancy, multiple"[MeSH Terms] OR "Twins"[MeSH Terms] OR "twinning, embryonic"[MeSH Terms] OR ((("Twins"[MeSH Terms] OR "Twins"[All Fields] OR "twin"[All Fields]) AND "pregancy"[Title/Abstract]) OR "multiple pregnancy"[Title/Abstract] OR "Twins"[Title/Abstract] OR "twin study"[Title/Abstract] OR "multiple birth"[Title/Abstract])) AND "Fetal Weight"[MeSH Terms]) OR "Birth Weight"[MeSH Terms] OR "Gestational Age"[MeSH Terms]) AND ("Growth Charts"[MeSH Terms] OR ("Growth Charts"[MeSH Terms] OR ("growth"[All Fields] AND "charts"[All Fields]) OR "Growth Charts"[All Fields] OR ("growth"[All Fields] AND "chart"[All Fields]) OR "growth chart"[All Fields] OR (("Birth Weight"[MeSH Terms] OR ("birth"[All Fields] AND "weight"[All Fields]) OR "Birth Weight"[All Fields] OR "birthweight"[All Fields] OR "birthweights"[All Fields]) AND ("chart"[All Fields] OR "charted"[All Fields] OR "charting"[All Fields] OR "chartings"[All Fields] OR "charts"[All Fields])) OR (("foetal weight"[All Fields] OR "Fetal Weight"[MeSH Terms] OR ("fetal"[All Fields] AND "weight"[All Fields]) OR "Fetal Weight"[All Fields]) AND ("chart"[All Fields] OR "charted"[All Fields] OR "charting"[All Fields] OR "chartings"[All Fields] OR "charts"[All Fields])) OR (("weight s"[All Fields] OR "weighted"[All Fields] OR "weighting"[All Fields] OR "weightings"[All Fields] OR "weights and measures"[MeSH Terms] OR ("weights"[All Fields] AND "measures"[All Fields]) OR "weights and measures"[All Fields] OR "weight"[All Fields] OR "body weight"[MeSH Terms] OR ("body"[All Fields] AND "weight"[All Fields]) OR "body weight"[All Fields] OR "weights"[All Fields]) AND ("chart"[All Fields] OR "charted"[All Fields] OR "charting"[All Fields] OR "chartings"[All Fields] OR "charts"[All Fields])))) AND "chart"[Title/Abstract]

**SCOPUS:**

| #1 | twin pregnancy OR twins OR multiple pregnancy | 104033 |
| --- | --- | --- |
| #2 | growth chart OR birthweight chart OR birthweight OR fetal weight OR growth rate | 68300 |
| #3 | #1 AND #2 | 5864 |
| #4 | TITLE-ABS-KEY ( chart ) | 236554 |
| **#5** | **#3 AND #4** | **212** |

Full strategy:

( ( twin AND pregnancy OR twins OR multiple AND pregnancy ) AND ( growth AND chart OR birthweight AND chart OR birthweight OR fetal AND weight OR growth AND rate ) ) AND ( TITLE-ABS-KEY ( chart ) )

CINAHL (EBSCO): **552**

((((MH "pregnancy, twin+") OR (MH "pregnancy, multiple+") OR (MH Twins+) OR (MH "twinning, embryonic+") OR ((((MH Twins+) OR Twins OR twin) AND (TI pregancy OR AB pregancy)) OR (TI "multiple pregnancy" OR AB "multiple pregnancy") OR (TI Twins OR AB Twins) OR (TI "twin study" OR AB "twin study") OR (TI "multiple birth" OR AB "multiple birth"))) AND (MH "Fetal Weight+")) OR (MH "Birth Weight+") OR (MH "Gestational Age+")) AND ((MH "Growth Charts+") OR ((MH "Growth Charts+") OR (growth AND charts) OR "Growth Charts" OR (growth AND chart) OR "growth chart" OR (((MH "Birth Weight+") OR (birth AND weight) OR "Birth Weight" OR birthweight OR birthweights) AND (chart OR charted OR charting OR chartings OR charts)) OR (("foetal weight" OR (MH "Fetal Weight+") OR (fetal AND weight) OR "Fetal Weight") AND (chart OR charted OR charting OR chartings OR charts)) OR (("weight s" OR weighted OR weighting OR weightings OR (MH "weights and measures+") OR (weights AND measures) OR "weights and measures" OR weight OR (MH "body weight+") OR (body AND weight) OR "body weight" OR weights) AND (chart OR charted OR charting OR chartings OR charts)))) AND (TI chart OR AB chart)

EMBASE: **192**

('pregnancy, twin'/exp OR 'pregnancy, multiple'/exp OR 'twins'/exp OR 'twinning, embryonic'/exp OR 'multiple pregnancy':ti,ab OR twins:ti,ab OR 'twin study':ti,ab OR 'multiple birth':ti,ab) AND ('growth charts'/exp OR (growth AND charts) OR 'growth charts' OR (growth AND chart) OR 'growth chart' OR (('birth weight'/exp OR (birth AND weight) OR 'birth weight' OR birthweight OR birthweights) AND (chart OR charted OR charting OR chartings OR charts)) OR (('foetal weight' OR 'fetal weight'/exp OR (fetal AND weight) OR 'fetal weight') AND (chart OR charted OR charting OR chartings OR charts)) OR (('weight s' OR weighted OR weighting OR weightings OR 'weights and measures'/exp OR (weights AND measures) OR 'weights and measures' OR weight OR 'body weight'/exp OR (body AND weight) OR 'body weight' OR weights) AND (chart OR charted OR charting OR chartings OR charts))) AND chart:ti,ab

**COCHRANE:**

ID Search Hits

#1 ([mh "pregnancy, twin"] OR [mh "pregnancy, multiple"] OR [mh Twins] OR [mh "twinning, embryonic"] OR (pregancy:ti,ab) OR "multiple pregnancy":ti,ab OR Twins:ti,ab OR "twin study":ti,ab OR "multiple birth":ti,ab) 2273

#2 [mh "Fetal Weight"] OR [mh "Birth Weight"] OR [mh "Gestational Age"] 5454

#3 ((("growth chart" ) OR ("birthweight chart" )) OR ("fetal weight chart" )) OR ("weight chart" ) 166

#4 #1 AND #2 **152**
